# Supplementary material for: Internet Altruistic Behaviors in Adolescents: Roles of Attention-Deficit/Hyperactivity Disorder, Impulsiveness, and Perceived Social Support
Source: Behav Sci (Basel). 2024 May 22;14(6):433. doi: 10.3390/bs14060433 (PMC11201007; doi:10.3390/bs14060433)
Supplement: Supplementary file 1 [file behavsci-14-00433-s001.zip › behavsci-2983428-supplementary.pdf]

Supplemetnary Table S1 Factors related to the four domain s of Internet altruistic behaviors: Bivariable linear regression analysis

	Internet support		Internet guidance		Internet sharing		Internet reminders	
	B (SE)	*p*	B (SE)	*p*	B (SE)	*p*	B (SE)	*p*
Age	0.435 (0.127)	0.001	0.093 (0.079)	0.241	0.139 (0.088)	0.114	0.166 (0.059)	0.006
Sex[a]	0.158 (0.708)	0.823	1.364 (0.426)	0.002	0.545 (0.481)	0.257	0.372 (0.327)	0.256
ADHD	1.014 (0.545)	0.064	0.633 (0.333)	0.058	0.518 (0.372)	0.164	0.416 (0.253)	0.100
Time spent on the Internet	0.059 (0.015)	<0.001	0.031 (0.009)	0.001	0.033 (0.010)	0.001	0.019 (0.007)	0.007
Inability in planning and looking ahead	-0.180 (0.065)	0.006	-0.089 (0.040)	0.026	-0.056 (0.044)	0.210	-0.095 (0.030)	0.002
Lack of self-control and perseverance	0.156 (0.058)	0.008	0.102 (0.035)	0.004	0.126 (0.039)	0.001	0.045 (0.027)	0.094
Novelty-seeking and quick in decision making	0.209 (0.083)	0.012	0.164 (0.050)	0.001	0.133 (0.056)	0.018	0.044 (0.039)	0.250
Perceived family support	0.003 (0.052)	0.961	-0.007 (0.032)	0.834	-0.001 (0.035)	0.971	0.015 (0.024)	0.536
Perceived peer support	0.213 (0.077)	0.006	0.129 (0.047)	0.006	0.111 (0.052)	0.034	0.110 (0.035)	0.002

[a]: Girls were used as the reference. ADHD: attention-deficit/hyperactivity disorder.

Supplemetnary Table S2 Factors related to the four domain s of Internet altruistic behaviors: Stepwise multivariable linear regression analysis

	Internet support		Internet guidance		Internet sharing		Internet reminders	
	B (SE)	*p*	B (SE)	*p*	B (SE)	*p*	B (SE)	*p*
Sex[a]	–	–	1.454 (0.405)	<0.001	–	–	–	–
Time spent on the Internet	0.056 (0.015)	<0.001	0.030 (0.009)	0.001	0.029 (0.010)	0.004	0.023 (0.007)	0.001
Inability in planning and looking ahead	-0.288 (0.072)	<0.001	-0.153 (0.044)	0.001	–	–	–	–
Lack of self-control and perseverance	0.280 (0.064)	<0.001	0.163 (0.039)	<0.001	0.138 (0.041)	0.001	-0.079 (0.031)	0.012
Perceived peer support	0.216 (0.078)	0.006	0.148 (0.048)	0.002	0.174 (0.053)	0.001	0.088 (0.037)	0.019

[a]: Girls were used as the reference. ADHD: attention-deficit/hyperactivity disorder.
